# Fibroblast Growth Factor 21: Possible Role in Growth and Metabolic Development during Early Life, Nutritional Interactions and Biological Actions

**DOI:** 10.1016/j.advnut.2026.100713

**Published:** 2026-08-08

**Authors:** Jibran A Wali, José Manuel Ramos-Nieves, Aristea Binia, Eline M van der Beek

**Affiliations:** 1 Nestlé Institute of Health Sciences, Nestlé Research, Lausanne, Switzerland; 2 Department of Pediatrics, University of Groningen, University Medical Centre Groningen, Groningen, The Netherlands

**Keywords:** fibroblast growth factor-21, metabolism, nutrition, infant growth, protein, amino acids, carbohydrate, early protein hypothesis

## Abstract

Fibroblast growth factor 21 (FGF21) is a hormone produced mainly in the liver that plays a crucial role in systemic metabolic regulation. Its diverse metabolic functions include promoting glucose uptake in adipocytes, enhancing fat oxidation, and inducing thermogenesis. Studies in rodents have shown that treatment with FGF21 protects against diet-induced obesity and when overexpressed, can even extend lifespan. The relationship between FGF21 and dietary macronutrients is complex and bidirectional. Diets low in protein and high in carbohydrates, particularly simple sugars, strongly stimulate the production of FGF21. Evidence from rodent experiments shows that once secreted, FGF21 acts on the brain, stimulating protein intake and suppressing the preference for sugars. Compared with adults, the role of FGF21 in metabolic physiology and programming during early life is, however, less well understood. In human infants, linear growth is negatively correlated with circulating FGF21 concentrations. In mice, lipids present in breast milk stimulate the production of FGF21 after birth, possibly activating thermogenesis. In this narrative review, we delve into the topic of FGF21 and its significance for metabolic development and growth during early life, the nutritional regulation of FGF21, and its impact on macronutrient preferences. Given the strong link between protein intake and FGF21 signaling, we hypothesize that FGF21-mediated metabolic regulation could provide a mechanistic basis for the early protein hypothesis. This hypothesis posits that higher protein intake in infancy exacerbates weight gain and increases risk of cardiometabolic disease in later life. Here, we aim to contribute to a deeper understanding of FGF21 biology, highlighting its potential to enhance healthspan and longevity through nutritional modulation during early life.

**Statement of significance**

This review examines the bidirectional relationship between nutrition and fibroblast growth factor 21 (FGF21) by detailing how dietary protein, fat, and carbohydrates influence FGF21 levels in humans and animals and discusses how evidence from rodent studies indicates that FGF21 affects macronutrient preferences. The review provides, to our knowledge, for the first time, a comprehensive assessment of the evidence on FGF21 physiology and its nutritional regulation during early life, a critical period for metabolic programming. It is proposed that high protein intake in infancy may suppress FGF21 levels, potentially impacting long-term metabolic health and appetite regulation.

## Introduction

The vast majority of fibroblast growth factor 21 (FGF21) hormone is synthesized by the liver, with relatively small amounts produced in the adipose tissue [1,2]. In humans, the *fgf21* gene is located on chromosome 19 and encodes a protein with 208 amino acids [1]. The *fgf21* gene was cloned in the year 2000 and FGF21 was found to significantly stimulate glucose uptake in adipocytes in early in vitro experiments [3,4]. Further research revealed its role in regulating glucose and lipid metabolism, energy expenditure, and body weight [1,2]. In initial preclinical in vivo studies, FGF21 was found to be strongly induced by fasting; later work showed a more complex bidirectional relationship with nutrition, involving regulation of FGF21 expression by intake of nutrients and a key role of FGF21 in regulating macronutrient preferences [5]. FGF21 analogs and receptor agonists have shown potential in clinical trials as a therapeutic target for metabolic disorders such as obesity and fatty liver [[1], [2], [3], [4],^6^]. Here, we start by reviewing the major metabolic functions of FGF21, the regulation by nutrition, and its role in controlling intake of specific nutrients. We subsequently specifically discuss the evidence linking FGF21 to growth and metabolic programming in early life. Lastly, we explain how integrating the role of FGF21 in the “early protein hypothesis”—a concept explaining the link between high protein supply in infancy and its impact on the long-term risk of obesity and associated disorders—will facilitate a deeper understanding of the nutritional foundations of lifelong metabolic health.

## Metabolic Functions of FGF21

In vitro and in vivo preclinical studies have been vital in revealing the mechanisms of FGF21 actions on diverse cell types such as adipocytes, hepatocytes, neurons, and bone cells [3,4]. In addition, clinical trials of pharmaceutical FGF21 analogs (e.g., LY2405319, pegbelfermin, and PF-05231023) and receptor agonists (e.g., NGM313 and BFKB8488A), developed to address FGF21’s short half-life (0.5–2 h), have offered valuable insights into the similarities and differences in FGF21 actions between humans and rodents (Table 1) [3,4,7,8]. For each metabolic effect of FGF21, we will first discuss the findings from animal experiments, followed by a review of the evidence from human studies. In humans, findings are primarily in the form of associations derived from cohort data, whereas evidence of its effects is established through the use of FGF21 analogs and receptor agonists.

**TABLE 1:** Summary of key metabolic effects of FGF21 treatment in rodents compared with humans

Parameter	Rodents	Humans
Body weight	Robust weight ↓ in obese rodent models	Modest (∼2%–6%) weight ↓
Energy expenditure	↑ via increase in BAT activity and via WAT beiging	Not convincingly demonstrated
Glycemic status	↑ glucose uptake, ↓ hepatic glucose output	No significant effect on fasting glucose or HbA1c; modest ↓ fasting insulin
Circulating lipids	↓ triglycerides; ↓ cholesterol	↓ triglycerides; ↓ LDL cholesterol; ↑ HDL cholesterol
Liver	↑ fat oxidation and ketogenesis; ↓ de novo lipogenesis; reversal of steatosis	Strong ↓ liver fat in MASH; improvement of MASH histology
White adipose tissue	↑ glucose uptake; beiging (UCP-1 induction)	Limited direct evidence in clinical trials
Brown adipose tissue	↑ UCP-1 expression and thermogenesis; ↑ glucose uptake	Not demonstrated; limited BAT mass in adults limits translational relevance
Preference for dietary macronutrients	↓ sugar/sweet and alcohol intake; ↑ protein intake	↓ preference for carbohydrate/sweet taste; protein appetite effects not yet studied in detail

Abbreviations: ↑, increase; ↓, decrease; BAT, brown adipose tissue; HbA1c, Glycated hemoglobin A1c; FGF21, fibroblast growth factor 21; MASH, metabolic dysfunction-associated steatohepatitis; UCP-1, uncoupling protein 1; WAT, white adipose tissue.

In human trials, FGF21 treatment is administered either as an FGF21 analog or as an FGF21 receptor agonist.

### Glucose metabolism

**FIGURE 1:**
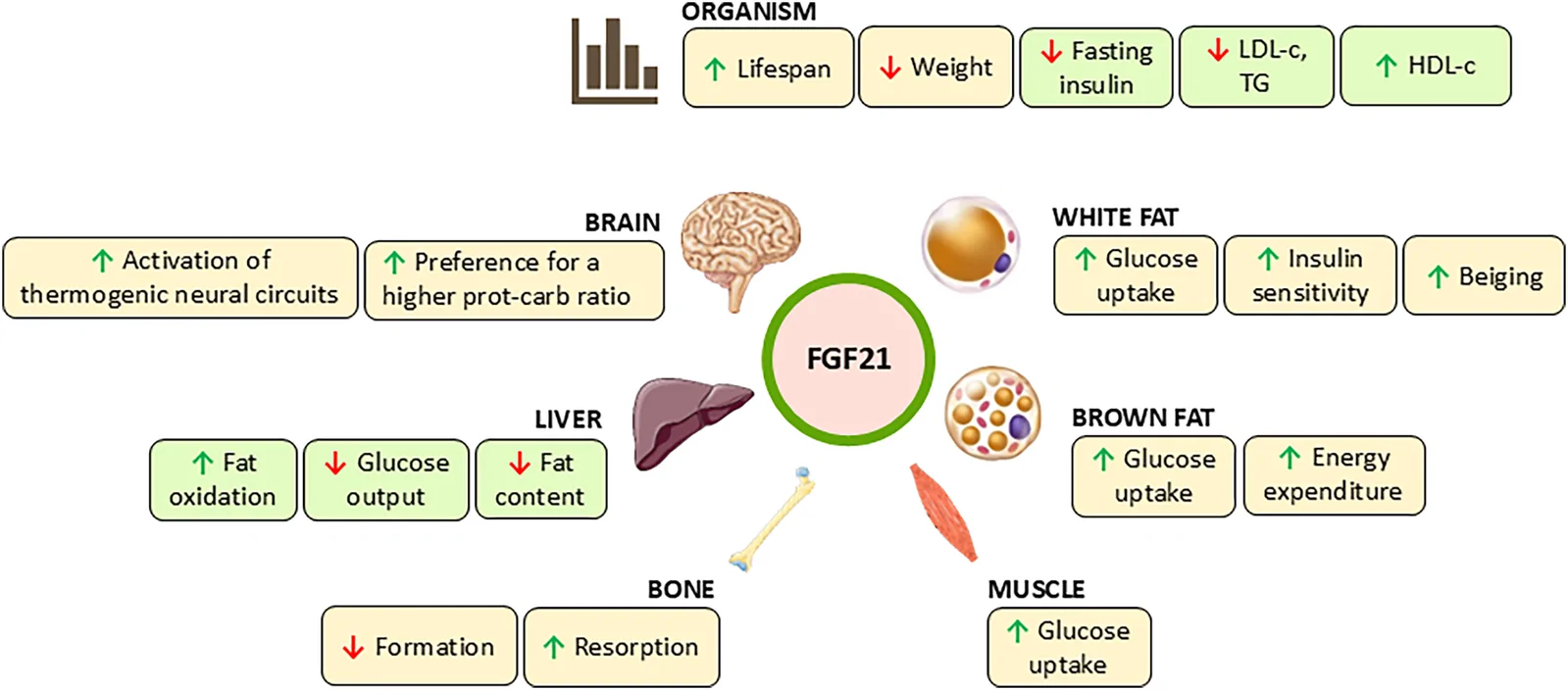
Major metabolic effects of fibroblast growth factor 21 (FGF21). FGF21 stimulates glucose uptake in peripheral tissues, improves insulin sensitivity, activates thermogenesis, increases fat oxidation, and regulates macronutrient preferences. In addition, overexpression of FGF21 in mice extends lifespan and mitigates diet-induced obesity. The yellow boxes represent metabolic effects observed in rodent studies, whereas the green boxes represent metabolic effects observed in rodents and reproduced in human clinical trials with pharmaceutical FGF21 agonists and analogs. Note that weight loss has not been consistently observed in clinical trials. Similarly, effects of pharmaceutical FGF21 agents on bone turnover have been inconsistent in clinical trials. Effects of FGF21 treatment on macronutrient preferences have not been well studied so far in humans. Yellow boxes: biological effects observed in rodent studies but not yet reproduced/investigated in human clinical trials with pharmaceutical FGF21-based agents. Green boxes: biological effects observed in rodents and already reproduced in human clinical trials with pharmaceutical FGF21-based agents. LDL-c, low-density lipoprotein cholesterol; HDL-c, high-density lipoprotein cholesterol; TG, triglycerides.

Preclinical studies conducted in mice, as well as in cultured hepatocytes and adipocytes, demonstrated that FGF21 is a potent and rapid insulin sensitizer [9,10]. It enhances glucose uptake in various tissues, including white (WAT) and brown adipose tissue (BAT) as well as skeletal muscle, improving glucose utilization and reducing blood glucose concentrations (Figure 1) [1,10]. FGF21 also contributes to glucose homeostasis by inhibiting hepatic glucose production [1,10]. In pancreatic islets, FGF21 improves function and survival of β-cells [11]. Moreover, FGF21 reduces inflammation in adipose tissue and liver, particularly reducing the number and activity of proinflammatory macrophages, leading to improved insulin sensitivity and insulin signaling [[12], [13], [14]].

#### Evidence from human studies

Contrary to the effects observed in rodents, pharmaceutical FGF21-based agents yielded only modest improvements in fasting glucose levels among individuals with type 2 diabetes, even though some clinical trials reported reductions in fasting insulin levels [[15], [16], [17]]. A meta-analysis of trials with FGF21 analogs reported no significant effect on fasting blood glucose or glycated hemoglobin levels [18]. However, fasting insulin concentrations in circulation were significantly lower in FGF21 analog-treated subjects, although this was not accompanied by significant improvement in HOMA index [18]. In line with this, a recent meta-analysis of clinical trials showed that FGF21 analogs produce a small but statistically insignificant reduction in fasting glucose and insulin concentrations [19].

### Lipid metabolism

FGF21 regulates lipid metabolism by increasing hepatic fat oxidation and ketogenesis (Figure 1) [20]. It also reduces the expression of genes associated with de novo lipogenesis in the liver [[20], [21], [22]]. In addition, treatment of obese mice with FGF21 reduces adiposity, liver steatosis, and circulating triglyceride concentrations [2,[20], [21], [22], [23]]. Mice lacking FGF21 showed reduced circulating ketone levels and decreased fat oxidation in the liver when compared with chow diet-fed wild-type mice [24]. Conversely, in transgenic mice overexpressing FGF21, ketogenesis significantly increased, and hepatic fat oxidation nearly doubled compared with wild-type mice [24]. In mice fed a high-fat diet (HFD), overexpression of FGF21 via tail vein *fgf21* plasmid injections reduced hepatic steatosis and decreased liver triglyceride content [25].

#### Evidence from human studies

Consistent with observations in mice, administering FGF21 analogs and agonists to overweight and obese adults has been shown to improve plasma lipid profiles. These benefits include reductions in triglycerides and LDL cholesterol concentrations and increases in circulating HDL cholesterol and 3-hydroxybutyrate concentrations [[15], [16], [17],[26], [27], [28]]. Opposite to the modest effects on glycemic markers, the impact of FGF21 mimetic agents on lipid profile is more pronounced, with improvements observed within just a few days [[15], [16], [17],[26], [27], [28]]. In addition, FGF21 analogs such as efruxifermin and pegozaferrin have shown remarkable improvement in reducing metabolic dysfunction-associated steatotic liver disease (MASLD) and associated steatohepatitis (MASH) in clinical studies [2,[29], [30], [31]]. For example, 12-wk efruxifermin treatment reduced hepatic fat content by ≤72% in subjects with MASH [31]. This has led to significant interest in developing FGF21-based novel therapeutic drugs for MASLD and MASH [6,8]. Moreover, dual FGF21 and glucagon-like peptide-1 agonists are also being investigated as therapeutic options for obesity and associated metabolic disorders [32,33].

### Energy expenditure, weight loss, and lifespan

Stimulation of energy expenditure plays a key role in the weight loss induced by FGF21. Although increasing glucose uptake in both WAT and BAT [1,4], FGF21 increases thermogenesis by inducing uncoupling protein-1 (Ucp-1) expression in BAT (Figure 1) [1]. Additionally, it promotes “beiging,” a process in which white adipocytes acquire characteristics of brown fat cells [1,23], further increasing thermogenesis and energy expenditure [1]. In mice made obese by HFD, treatment with FGF21 promoted weight loss and improved glucose tolerance [1,[21], [22], [23]]. These changes were accompanied by an increase in glucose uptake in WAT and BAT and a striking increase in energy expenditure [1,21,22]. Similarly, transgenic mice overexpressing FGF21 were leaner, showed improved insulin sensitivity compared with wild-type mice, and were resistant to HFD-induced-obesity [1,[21], [22], [23],^34^,^35^].

Interestingly, mice with high FGF21 expression have a disrupted liver growth hormone (GH)/insulin-like growth factor (IGF)-1 axis and a longer lifespan and healthspan than control mice [36,37]. The lifespan and metabolic phenotype of FGF21 transgenic mice is similar to that of the Ames dwarf mice that have lower levels of GH because of an autosomal recessive mutation in prophet of Pit-1 (Prop1) gene. They are smaller in size and body weight but have improved markers of metabolic health during aging and substantially longer lifespan when compared with their wild-type littermate controls [38]. In contrast to FGF21 transgenic mice, FGF21-deficient mice are obese and insulin resistant [39]. Intriguingly, FGF21 levels are elevated in HFD-fed wild-type mice and leptin-deficient *ob/ob* obese mice [23,40]. This has led to the suggestion that obesity may give rise to an FGF21-resistant state [23,40]. Treatment of mice with higher doses of FGF21 is able to overcome this resistance and is effective in activating receptor signaling [1]. Moreover, it was found that HFD-fed and *ob/ob* mice were more sensitive to glucose-lowering effects and weight loss induced by exogenous FGF21 than lean mice [41]. These findings indicate the possibility that an increase in FGF21 levels could be a compensatory response to mitigate the obesity-related decline in insulin sensitivity [42]. The increase in expression and levels of FGF21 by hepatocytes has also been described as a compensatory response to various forms of metabolic stress, such as starvation, fasting, steatosis, viral infections, and cellular injury [43,44]. At the organelle level, an increase in FGF21 has been identified as a response to endoplasmic reticulum (ER) stress [45]. In mice, it was observed that ER stress induced in liver cells through tunicamycin injections resulted in an increase in FGF21 expression [45]. However, this effect was diminished in mutant mice where the phosphorylation of the ER stress pathway factor, eukaryotic translation initiation factor-2α, was blocked in hepatocytes [45]. Besides ER stress, mitochondrial stress and diseases caused by genetic defects in the mitochondrial oxidative phosphorylation machinery have also been shown to increase FGF21 expression [46,47].

#### Evidence from human studies

Fasting FGF21 concentrations correlate positively with BMI in human adults [48], and FGF21 levels are elevated in obese humans [23,40], indicating a compensatory response to metabolic stress and insulin resistance. In clinical studies, the treatment of overweight and obese subjects with FGF21 analogs such as LY2405319 and PF-05231023 for 4 to 5 wk led to 2% to 6% loss in body weight [15,16]. However, trials involving other analogs such as Pegbelfermin and Efruxifermin did not induce significant weight loss [17,27], and only a transient weight loss was induced by a single dose of long-acting FGF21 agonist antibody BFKB8488A [28]. Evidence from nonhuman primates and human subjects indicates that the relatively modest weight loss induced by FGF21-based drugs may be a consequence of reduced food intake [16,28].

### Sex-dependent metabolic effects of FGF21

Differences in FGF21 physiology between males and females have been observed in rodent studies [49]. Generally, male rodents exhibit a more pronounced increase in circulating FGF21 levels than females when fed obesogenic diets [49]. For example, male rats demonstrated a significant upregulation of hepatic FGF21 protein expression in response to a high-fat, high-fructose diet (HFFD), a response not observed in females [50]. This aligns with observations that male mice are more susceptible to HFD-induced metabolic disturbances [51], as well as data showing that obese male mice are more responsive to FGF21 treatment—exhibiting greater weight loss and reduced hepatic fat content than their female counterparts [52].

There is also preclinical evidence linking FGF21 to the metabolic changes associated with menopause. For example, FGF21 plays a crucial role in mediating the metabolic effects of estrogen therapy in ovariectomized rodent models of menopause. In rats, ovariectomy reduced FGF21 levels and exacerbated HFFD-induced hepatic steatosis, whereas estrogen treatment increased FGF21 levels and alleviated steatosis in these animals [50]. In ovariectomized mice, estrogen was found to upregulate *fgf21* transcription via activation of hepatic estrogen receptor-α [53]. Estrogen treatment also increased energy expenditure; an effect lost in FGF21-deficient ovariectomized mice [53]. Another study demonstrated that FGF21 is essential for WAT browning and reduced subcutaneous fat mass in ovariectomized mice treated with exogenous estrogen [54]. Liver-specific FGF21 deficiency abolished estrogen-induced WAT browning in these mice [54].

#### Evidence from human studies

In humans, the role of sex in FGF21 biology is less well studied and understood, with inconsistent findings necessitating further research. This is exemplified by the difference across studies in fasting FGF21 levels. A study reported that healthy adult females had higher fasting serum FGF21 levels and elevated circulating FGF21 concentrations in response to high-fructose feeding [55]. However, another study found no significant difference in fasting serum FGF21 levels between adult males and females, and FGF21 concentrations were comparable between the sexes after a glucose bolus [56].

In females, the relationship between menopause and FGF21 is confounded by the positive correlation between BMI and FGF21 levels. A study found that FGF21 concentrations were ∼2.5-fold higher in perimenopausal females with overweight and obesity with metabolic syndrome than in those with normal BMI, and FGF21 levels showed a significant positive correlation with waist-to-hip ratio [57]. In a cohort of females (mean age: 57 y; SD: 13 y), besides BMI, serum FGF21 concentrations were positively associated with carotid atherosclerosis [58]. This menopause-related increase in FGF21 could be attributed to increasing BMI and FGF21 resistance or, as others have suggested, to deficient protein intake relative to increased postmenopausal protein requirements [59]. It has been proposed that increasing protein intake by 0.1 to 0.2 g/kg/d while reducing daily energy intake by 5% to 10% (compared with baseline) could be an effective strategy to counter metabolic impairments and improve FGF21 levels in postmenopausal females [59].

Studies on circulating FGF21 concentrations in females with gestational diabetes mellitus (GDM) have been inconsistent, with reports of both higher and lower FGF21 levels compared with females with normal glucose tolerance (NGT) [60]. For example, in 1 study, serum FGF21 concentrations in females with GDM were ∼50% of those in NGT mothers [61]. Conversely, Megia et al. [62] found that circulating FGF21 levels were higher in females with GDM than in those with NGT. Interestingly, children aged 9 to 16 y, born to mothers with GDM, exhibited ∼31% lower serum FGF21 levels compared with those born to non-GDM mothers, with an inverse association observed between their FGF21 concentrations and body fat percentage [63].

### Bone remodeling

An important aspect of FGF21 biology with implications for growth and mobility pertains to its effects on skeletal biology. Rodent studies suggest that exposure to high levels of FGF21 reduces bone mass (Figure 1) [2]. Mice overexpressing FGF21, for example, showed shorter body and tibial lengths compared with wild-type controls [34], as well as significantly lower trabecular bone mass and bone mineral density [64]. Similarly, in lactating C57BL/6 mice, there was a 2.4-fold increase in serum FGF21 concentration on day 7 of lactation, which was accompanied by increased bone resorption and 10% reduction in bone mineral density [65]. Obese mice treated with recombinant FGF21 for 2 wk displayed reduced bone formation and increased bone resorption [64]. Conversely, FGF21 deletion resulted in greater bone mass than in wild-type mice [64]. Another study found that FGF21 knockout mice under calorie restriction had longer body and bone lengths than wild-type controls [66]. However, contrary to these findings, a later study showed that the treatment of HFD-fed obese mice with recombinant FGF21 does not induce bone loss or reduce bone mineral density, and bone morphology in FGF21-deficient mice was not different from that in wild-type mice [67]. The reasons for the inconsistent findings among these studies remain unclear; however, they may be attributed to life stage and diet-associated variations in FGF21 levels and the duration of exposure to elevated concentrations of FGF21.

#### Evidence from human studies

Studies examining the association between FGF21 levels and markers of bone health in humans have also yielded inconsistent results [68]. For example, a study in postmenopausal females found that serum FGF21 concentrations were negatively associated with hip geometry and mechanical strength [69]. In contrast, another study in Chinese postmenopausal females reported a positive association between circulating FGF21 levels and lumbar bone mineral density [70]. In human trials with FGF21 analogs, PF-05231023 adversely affected markers of bone turnover (e.g., reduced osteocalcin levels), but other FGF21 analogs did not have negative effects on markers of bone health [16,27,71]. It has been speculated that the decline in bone mass could be secondary to the weight loss induced by the PF-05231023 treatment [2].

### Molecular signaling

A combination of experiments in cultured cells and in rodent models has revealed mechanisms and signaling pathways through which FGF21 affects metabolic physiology. Direct metabolic actions of FGF21 are confirmed by findings from studies on the metabolic phenotypes of mice that either lack or overexpress FGF21. For example, chow diet-fed mice deficient in FGF21 exhibit impaired glucose tolerance, reduced energy expenditure, and lower levels of ketones [24,39]. In contrast, mice that overexpress FGF21 show lower fasting glucose levels and increased energy expenditure when fed a chow diet [35,72]. The cellular signaling that mediates these physiological effects of FGF21 involves a complex process that begins with the binding of FGF21 to its receptor, FGFR1c (fibroblast growth factor receptor 1c) [1,20]. Upon FGF21 binding, FGFR1c undergoes a conformational change and recruits transmembrane coreceptor β-Klotho, which is essential for FGF21 signaling [4,73]. The extracellular domain of β-Klotho facilitates physical interaction between FGF21 and its receptor [4]. The importance of β-Klotho is confirmed by the observation that mice lacking β-Klotho were unresponsive to the metabolic effects of FGF21 treatment [74,75]. FGFR1c is widely expressed but β-klotho expression is restricted to WAT, BAT, liver, and pancreas [10]. β-klotho expression has also been reported in the area postrema, nucleus tractus solitarii, suprachiasmatic nucleus, and the paraventricular nucleus of the brain [2,10]. This expression pattern of β-klotho confers tissue specificity for the actions of FGF21 [2,10]. Activation of the FGF21-FGFR1c-β-Klotho complex causes phosphorylation of FGFR substrate 2α (FRS2α) and activation of downstream signaling pathways including the mitogen-activated protein kinase (MAPK)/extracellular signal-regulated kinase and the phosphoinositide 3-kinase/protein kinase B pathways [2,20,73]. These signaling cascades induce changes in target gene expression and regulate various cellular processes, such as glucose uptake, lipid metabolism, and energy balance [73,76]. In addition, FGF21 signaling in the brain mediates the metabolic responses to intake of dietary nutrients and their deprivation [2].

An important aspect of FGF21 signaling in target tissues, relevant to its effects on growth and metabolic regulation, is its interaction with mechanistic target of rapamycin (mTOR) activity. This relationship has been investigated primarily in cultured cells and mouse models. In hepatocytes, FGF21 suppresses mTOR phosphorylation, an effect that led to improved hepatic insulin sensitivity and enhanced glycogen synthesis [77]. In contrast, in cultured adipocytes, FGF21 has been shown to activate mTOR via MAPK, leading to increased adiponectin secretion, induction of UCP1 expression, and stimulation of glucose uptake, without impairing insulin sensitivity [78]. These metabolic effects of FGF21 were dependent on mTOR activity, as they were suppressed by the mTOR inhibitor rapamycin [78].

## Crosstalk between Macronutrient Intake and FGF21

This section explores the impact of both the quantity and type of dietary macronutrients—namely, protein, fat, and carbohydrates—on circulating concentrations of FGF21. In this context, FGF21 can be considered a biomarker for the intake of various dietary macronutrients, with supporting evidence derived from preclinical experiments, human trials with nutritional interventions, and data from human cohorts that demonstrate associations between the intake of specific macronutrients and circulating FGF21 concentrations. We will also discuss how FGF21 levels directly influence preferences for macronutrients. The direct evidence for these preferences principally comes from rodent studies, whereas human evidence is mostly limited to associations between single-nucleotide polymorphisms and the intake of particular nutrients.

### Carbohydrates and sugars

#### High-carbohydrate diets and FGF21

**FIGURE 2:**
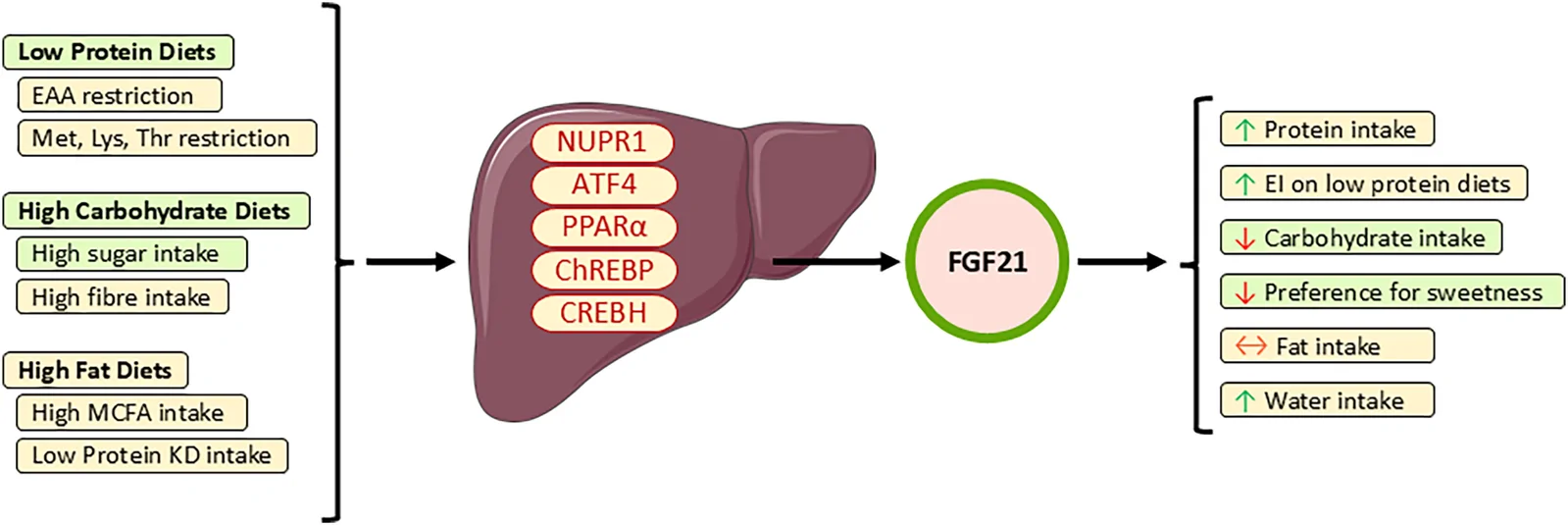
Nutrients—fibroblast growth factor 21 (FGF21) crosstalk. Dietary nutrients induce FGF21 synthesis in the liver via transcription factors PPARα, ATF4, ChREBP, CREBH, and NUPR1. Higher levels of FGF21 alter macronutrient intake and preferences via its actions in the brain. This includes stimulation of protein and water intake, suppression of carbohydrate intake, and reduced preference for sugars. FGF21 has not been shown to directly affect fat intake. The yellow boxes represent metabolic effects observed in rodent studies, whereas the green boxes represent metabolic effects observed in rodents and reproduced in human clinical trials. Impact of FGF21-based pharmaceutical agents on nutrient preferences is not well studied so far in humans. ATF4, activating transcription factor 4; ChREBP, carbohydrate-responsive element-binding protein; CREBH, cyclic adenosine monophosphate (cAMP)-responsive element-binding protein H; EAA, essential amino acids; EI, energy intake; KD, ketogenic diet; Lys, lysine; MCFA, medium-chain fatty acids; Met, methionine; NUPR1, nuclear protein 1; PPARα, peroxisome proliferator-activated receptor α; Thr, threonine. Yellow boxes: biological effects observed in rodent studies but not yet reproduced/investigated in human clinical trials. Green boxes: biological effects observed in rodents and already reproduced in human clinical trials.

FGF21 responds to and regulates carbohydrate intake by integrating central and peripheral signals for metabolic homeostasis (Figure 2) [2,20]. In mice, both acute and chronic high-carbohydrate diets stimulate FGF21 production and secretion from the liver [5]. This induction of FGF21 occurs across various carbohydrate types, including starch, sucrose, high-fructose corn syrup, glucose, and fructose [79,80]. In long-term (18-wk dietary intervention) mouse experiments, a relatively similar increase in plasma FGF21 concentrations was observed with high glucose compared with high fructose intake [79,80]. Additionally, high intake of fermentable fibers raises circulating FGF21; for example, diets rich in resistant starch resulted in a 5-fold increase over isocaloric native starch-rich diets [79,81]. The carbohydrate-induced increase in hepatic FGF21 production is mediated via transcription factors carbohydrate-responsive element-binding protein and peroxisome proliferator-activated receptor α (PPARα) [5,10].

Characteristic of a negative feedback loop, FGF21 acts on the hypothalamus to suppress the desire for sweet foods and drinks [2,5,20]. Von Holstein-Rathlou et al. [82] showed that FGF21 deficiency in mice led to increased consumption of high-sucrose diets, whereas FGF21 overexpression or treatment decreased this intake. Concurrently, Talukdar et al. [83] found that genetically or therapeutically increasing FGF21 levels reduced sucrose and also alcohol intake. Increased FGF21 also lowered intake of noncaloric sweeteners like saccharin and sucralose, with a stronger effect observed for noncaloric sweeteners compared with sucrose [82,83]. FGF21 did not acutely affect intake of less-sweet carbohydrates like maltodextrin or lactose, suggesting an effect specific for a high level of sweetness [82].

#### Evidence from human studies for a carbohydrate–FGF21 relationship

Consistent with rodent studies, human trials have shown an acute increase in plasma FGF21 levels after intake of simple sugars [82,84]. Both healthy individuals and subjects with metabolic syndrome showed FGF21 spikes after consuming 75 g glucose or fructose, with fructose causing a rapid but brief rise and glucose a slower, sustained increase [84]. Subjects with metabolic syndrome showed a greater FGF21 response than healthy participants [84]. Additionally, dextrose infusion-induced hyperglycemia tripled circulating FGF21 levels in healthy participants [82], and 3 d of high dietary carbohydrate intake raised FGF21 in healthy males [85].

Similar to FGF21 treatment-induced suppression of intake of sugar solutions in mice [82,83], a single dose of long-acting FGF21 agonist antibody, BFKB8488A, reduced calorie intake, significantly reduced the proportion of carbohydrate in the diet (at the expense of small increases in protein and fat percentages) and reduced preference for sweet foods in adults with overweight and obesity [28]. These findings indicate a conserved FGF21–carbohydrate relationship across species.

Genome-wide association studies (GWAS) have linked variants in the FGF21 locus with macronutrient intake and sugar preference in humans [10,86]. Variants like rs838133 and rs838145 are associated with increased carbohydrate intake and preference for sweets [[86], [87], [88]]. For example, in the United Kingdom Biobank dataset, the rs838133 minor allele A associated with higher carbohydrate intake, blood pressure, and waist-to-hip ratio [88]. Although the function of this allele is not characterized, it is probably associated with lower FGF21 expression as it is associated with increased sugar intake, mirroring findings in FGF21 knockout mice [82,83].

### Fats and ketogenic diets

#### High-fat diets, ketogenic diets, and FGF21

The impact of fat intake on FGF21 levels has been widely studied in the context of high-fat, very low-carbohydrate ketogenic diets (KDs) (Figure 2), yet the role of dietary fat in FGF21 physiology remains unclear. Mouse studies show that KDs significantly induce FGF21 [2,10]. For example, mice fed a KD [79% fat, 10% protein, and 1% carbohydrate (79% F, 10% P, 1% C)] showed a >25-fold increase in hepatic FGF21 messenger RNA (mRNA) expression after 3 and 30 d [89]. KD increases FGF21 expression via PPARα, as *fgf21* induction was blocked in PPARα-deficient mice [89]. The increase in FGF21 expression with KDs has been described as crucial for inducing ketosis and mediating various metabolic effects of KDs [2,10]. KD-fed mice with adenoviral *fgf21* knockdown displayed reduced ketone levels, higher hepatic triglyceride content, and increased serum triglycerides. Furthermore, KD-fed FGF21-deficient mice had reduced expression of mitochondrial activity and biogenesis-regulating factors peroxisome proliferator-activated receptor γ coactivator 1 α and peroxisome proliferator-activated receptor γ coactivator 1 β, and increased activation of lipogenic transcription factor sterol regulatory element-binding protein 1c in the liver [39]. Interestingly, the protein content of KDs has been shown to significantly influence FGF21 levels; mice on a 5% protein KD had higher ketosis and showed a ∼15-fold increase in FGF21 concentrations (compared with control diet) but FGF21 levels did not increase on a 13% protein KD [90]. This effect was replicated in rats, where a 5% protein KD raised ketones and FGF21 levels, while adding 1 g extra protein per 10 g of KD reduced FGF21 levels in mice without affecting ketonemia [91]. This raises questions about the necessity of FGF21 for inducing ketosis, but it suggests that the balance of dietary protein and fat in KDs is crucial for achieving FGF21-dependent metabolic benefits.

In addition to KDs, FGF21 levels increase in mice fed HFDs that are rich in saturated fats [23,40]. However, these energy-dense diets often contain added sucrose, have a lower percentage of protein energy, and may also be lower in essential fatty acid content compared with control diets [[92], [93], [94], [95]]. This begs the question whether elevated FGF21 levels are due to high fat content or due to changes in the balance of other nutrients or a consequence of fat quality [2,40]. Indeed, a study using isocaloric diets with fixed 20% protein found that replacing carbs with fat lowered fasting FGF21 concentrations [80]. Additionally, the type of fat in HFDs matters: mice fed a medium-chain fatty acid (MCFA)-rich HFD (from caprylic and capric triglycerides) for 5 wk had reduced liver fat, lower plasma triglycerides, and improved glucose tolerance compared with long-chain fatty acid-fed mice (from lard and soybean oil) [96]. A single dose of MCFA was enough to produce a 4-fold increase in FGF21 levels by activating the transcription factor cyclic adenosine monophosphate-responsive element-binding protein H (CREBH) [96]. In FGF21-deficient mice, these MCFA benefits on hepatic steatosis were reduced [96].

In contrast to the reduced carbohydrate intake in mice, fat consumption was not affected by FGF21 treatment [82,97]. Similarly, in macronutrient-choice tests, FGF21 affected carbohydrate and protein intake but not fat [97], and both FGF21 knockout and overexpressing mice consumed similar amounts of a lipid solution [82]. Overall, FGF21 does not influence preference for fat-rich foods; however, its production varies based on dietary fat content, fat-protein and fat-carbohydrate ratio, and type of fat. The role of essential fatty acids in FGF21 physiology remains unclear.

#### Evidence from human studies for a fat–FGF21 relationship

Dietary trials show that, unlike mice, KDs do not increase FGF21 production in humans. For example, a 3-mo KD regimen in participants with obesity resulted in 9% weight loss but paradoxically produced a 42% decline in FGF21 concentrations (compared with baseline) [98]. In the same study, treatment of participants with obesity with a PPARα agonist for 2 wk increased plasma FGF21 by 39%, reduced fasting glucose, cholesterol, and triglyceride concentrations, and significantly decreased liver fat, raising doubts on the conclusion from rodent studies that KDs could induce *fgf21* expression via PPARα [98]. Similarly, in another study, a 12-d KD intervention in adults with BMI ranging from 19 to 27 kg/m^2^ had no effect on circulating FGF21 concentrations despite the induced ketosis and reduced fasting insulin concentrations [48]. Exposure to a KD also did not alter serum FGF21 levels in epileptic children [99]. It is unclear why KDs lead to high FGF21 levels in mice, but not in humans. A possible explanation could be that KDs used in mice often have a lower protein content (∼10%) because ketosis is otherwise minimal at a standard level of dietary protein (∼18%–20%), but in humans, KDs tend to have a higher protein content (∼20%–35%) compared with habitual diets [93,100]. Other factors include differences in protein requirements and possible differences in the capacity to mobilize muscle protein to fuel gluconeogenesis. It will be worth exploring if, as in mice [96], MCFAs and possibly other bioactive lipids could induce FGF21 production in humans. The impact of increasing circulating FGF21 concentrations on dietary fat preference is yet to be explored. However, findings from GWAS indicate that the FGF21 variants (rs838133 and rs838145) associated with higher carbohydrate intake are also linked to lower fat intake [86,87,101].

### Proteins and amino acids

#### Low-protein diets and FGF21

Contrary to the increase in FGF21 expression induced by high-carbohydrate diets, data from rodent experiments show that reducing dietary protein content (and replacing it with either fat or carbohydrate) increases circulating FGF21 concentrations (Figure 2). Mice and rats fed 4% to 9% protein (% energy) diets exhibit a substantial rise in their circulating FGF21 levels (∼6–8-fold compared with control diets) [91]. The evidence that high-carbohydrate diets, KDs, HFDs, and low-protein diets all induce FGF21 expression indicates a role for macronutrient interactions in regulating FGF21 expression. For example, the protein-to-carbohydrate ratio, much like the protein-to-fat ratio in KDs, significantly impacts FGF21 production. In a study, mice were fed isocaloric diets with varied protein and carbohydrate levels—low protein–high carbohydrate (5% P, 84% C), normal protein–normal carbohydrate (14% P, 75% C), or high protein–low carbohydrate (54% P, 35% C)—with fat fixed at 11% [102]. After 3 wk, hepatic *fgf21* expression was the highest in the low-protein–high-carbohydrate group and the lowest in the high-protein–low-carbohydrate group [102]. Solon-Biet et al. [103] applied “nutritional geometry methodology” to analyze FGF21 production in mice by varying protein, fat, and carbohydrate levels across 25 diets at different energy densities. It was found that protein intake had the strongest effect, with maximum plasma FGF21 concentrations observed on low-protein–high-carbohydrate diets with minimal effects of dietary fat content [103]. Further studies showed that this effect was also responsive to carbohydrate type and protein source [79,80,104].

The metabolic effects of low-protein rodent diets mediated by FGF21 depend on the degree of protein restriction. Reducing dietary protein content from the standard 18% to 20% (AIN93G diet) down to 7% to 10% increases FGF21 and energy expenditure but leads to weight gain because of increased energy intake in mice [79,100,104,105]. However, very low-protein diets (4%–6% P) strikingly increase FGF21 levels and thermogenesis, promote weight loss, resulting in leaner mice despite high energy intake [79,104,105]. FGF21-deficient mice fed 4% to 5% protein diets lack this metabolic phenotype, showing higher liver triglycerides, cholesterol, and plasma triglycerides [91,106]. FGF21 signaling in the brain is crucial for these metabolic effects, as β-Klotho deletion in the brain reverses these effects [107]. In addition, there is a substantial FGF21-stimulated increase in water intake in mice fed very low-protein diets [79,108]. Going beyond short- to medium-term metabolic effects, a study in 3-mo-old male mice fed either 4% or 18% protein diets (isocaloric) showed that the 4% protein diet extended lifespan and decreased frailty at 22 mo of age, with these benefits diminished in FGF21-deficient mice [109]. The metabolic effects of dietary protein restriction are sex-dependent, being more pronounced in males [110]. Compared with males, female mice experienced a less marked decrease in body weight, fat mass, and plasma triglyceride levels, accompanied by a relatively smaller (∼50% less) increase in plasma FGF21 concentrations after 30 d of exposure to a 4% protein diet [110].

Hepatic FGF21 production is transcriptionally regulated by protein intake. Low-protein-induced FGF21 increases are reduced in mice lacking PPARα or the serine/threonine kinase general control nonderepressible 2 (GCN2) [91]. GCN2 activation leads to downstream binding of transcription factor activating transcription factor 4 (ATF4) to the FGF21 promoter to induce its expression [111,112]. Recent evidence suggests that microbiota-derived ammonia is a critical mediator linking low-protein diets to hepatic FGF21 expression [113]. Others have reported that the transcriptional regulator nuclear protein-1 (NUPR1) also regulates FGF21 expression in mice fed protein-restricted diets [114].

#### Amino acid restriction and FGF21

In addition to protein quantity, evidence from rodent experiments shows that the type of protein, especially its essential amino acid profile, influences FGF21 levels. For example, protein-restricted diets induce higher FGF21 levels in mice when protein is sourced from casein rather than whey [104]. Whey is more quickly digested than casein [115]. Compared with whey, casein contains relatively higher levels of amino acids such as phenylalanine and glutamic acid, while having lower levels of amino acids such as leucine and cysteine [116]. Reducing protein content in mouse diets from 33% to 13% resulted in increased food intake and weight gain, with a more pronounced effect observed in casein-based diets compared with whey-based diets [104]. When given a choice, mice preferred whey over casein as the overall dietary protein content decreased and plasma FGF21 concentrations increased [104]. Analyzing intakes of essential amino acids showed that this increasing whey-to-casein intake ratio helped maintain stable levels of cysteine, threonine, and tryptophan ingestion. Methionine, a cysteine precursor, was supplemented in these diets to prevent its deficiency as per the standard practice for rodent diets [100,104].

Dietary restriction of certain essential amino acids—particularly methionine, lysine, and threonine—strongly elevates circulating FGF21 and yields metabolic benefits. Severe restriction of these amino acids (>65% restriction compared with control diets) markedly increases FGF21, whereas restriction of branched-chain amino acids (BCAAs), such as leucine, results in smaller increases in FGF21 [[117], [118], [119], [120], [121]]. For example, the study by Yap et al. [122] showed that mice on a 5% protein diet for 3 wk had 3 times higher serum FGF21 concentrations, increased energy expenditure, and improved insulin sensitivity compared with those on an 18% protein diet. These effects were replicated by reducing essential amino acids to levels found in a 5% protein diet while adding nonessential amino acids to match the total amino acid content of the 18% protein diet. Further experiments revealed that reduced levels of lysine, threonine, tryptophan, and methionine, rather than BCAAs, primarily drove the increase in FGF21 and associated metabolic benefits [122]. In another study, methionine restriction alone raised FGF21 by 7-fold within 48 h, improving glucose tolerance without weight loss [123]. Extended restriction for 8 wk raised FGF21 levels to ∼8000 pg/mL, compared with <1000 pg/mL in control diets, inducing weight loss and reducing hepatic lipogenesis [123]. Studies with FGF21-deficient mice confirm that these metabolic effects of methionine restriction are dependent on FGF21 signaling [124,125]. Restriction of BCAAs such as leucine raises FGF21 but less significantly (e.g., ∼3700 pg/mL on a leucine-restricted diet) and has weaker effects on glucose tolerance and body weight than methionine restriction [118,121,123]. Additionally, lysine or threonine restriction in rats sharply elevated FGF21 (∼30,000 pg/mL compared with <3000 pg/mL in controls), driving weight loss despite increased energy intake [120]. These findings underscore that restricting methionine, lysine, and threonine is more effective than BCAA restriction for enhancing FGF21 and supporting a favorable metabolic profile.

#### Stimulation of protein intake by FGF21

In contrast to suppressing carbohydrate intake, FGF21 was found to promote protein intake and encourage a preference for high-protein diets in rodents. In food choice experiments, mice given access to isocaloric low-protein (4% P, 74% C, 22% F) and high-protein (31% P, 47% C, 22% F) diets showed that 3 d of intracerebroventricular FGF21 injections significantly increased preference for the 31% protein diet, boosting protein intake without affecting total energy intake [107]. Recently, it was reported that although FGF21 mainly drives protein appetite, its effects on calorie intake and sweet preference depend on dietary context [126]. In mice offered diets with high or low protein–carbohydrate ratios (35% compared with 5% P), treatment with an FGF21 pump for 7 d increased protein and energy intake but kept carbohydrate intake stable. The increase in protein intake was greater if mice were preexposed to the 35% (compared with 5%) protein diet, suggesting a memory effect [126]. FGF21 also more effectively reduced sucrose drink intake when mice consumed a higher-carbohydrate diet, showing that FGF21’s impact on nutrient intake and sweet preference depends on the background diet [126].

#### Evidence from human studies for a protein–FGF21 relationship

Evidence from short-term clinical studies supports the link between low protein-high carbohydrate intake and elevated FGF21 concentrations [91,119,127]. For example, a 4-d ad libitum diet containing 10% protein led to a 14% increase in daily caloric intake and a 6-fold rise in plasma FGF21 levels compared to a 25% protein diet in healthy participants [128]. Similarly, males with obesity consuming a 7% to 9% protein diet for 6 wk experienced a 2-fold increase in plasma FGF21 compared with their habitual diet [119]. In another study, subjects overfed by 40% of baseline energy on a 5% protein diet exhibited a 171% increase in plasma FGF21 concentrations (compared with baseline) after 28 d, whereas subjects on a 15% protein diet showed no significant change [91]. A very low-protein diet (3% P) increased circulating FGF21 levels in healthy subjects by 3-fold after just 24 h, and FGF21 levels were positively correlated with energy expenditure [129]. Importantly, subjects with a smaller increase in FGF21 after this diet challenge gained more weight after 6 mo in free-living conditions, indicating that diminished FGF21 response is linked to the “thrifty” phenotype [129]. Ramne et al. [130] observed that protein intake had a more substantial impact on FGF21 levels than carbohydrates or fat. Their study demonstrated that the incremental AUC (iAUC) for postprandial plasma FGF21 after a 75 g sucrose drink was 484 pg/mL, which dropped to −35 pg/mL when 100 kcal of protein (25 g protein + 75 g sucrose) was added, whereas adding 100 kcal of fat (11 g fat + 75 g sucrose) did not significantly alter the iAUC compared with the sucrose-only drink [130]. Consistent with the findings of acute clinical trials, data from the United Kingdom Biobank cohort showed that higher plasma FGF21 levels were associated with increased alcohol and free sugar consumption and decreased intake of protein, complex carbohydrates, and fat [131]. This suggests that the relationship between macronutrients and cross-sectional plasma FGF21 levels in the cohort is more indicative of the impact of macronutrient intake from habitual diets on plasma FGF21, rather than reflecting the influence of FGF21 on macronutrient preferences [131]. Taken together, evidence from human and rodent studies suggests that protein is the primary nutrient regulating circulating FGF21 concentrations.

An important consideration when comparing the effects of dietary protein intake on circulating FGF21 levels in humans and rodents is the difference in their protein requirements. In humans, the recommended protein intake to maintain nitrogen balance is ∼0.8 g/kg body weight [132], and habitual diets in Western nations typically provide ∼15% to 16% of total energy from protein [133]. In contrast, standard mouse diets contain ∼18% to 20% protein during the growth phase and ∼14% protein for the maintenance phase, with adult mice consuming ∼2 to 5 g of food per day [79,100,134,135]. This corresponds to a markedly higher protein intake on a g/kg body weight basis in mice than in humans. As a consequence, reducing dietary protein from standard to low-protein (4%–5% P) diets in mice represents a more severe relative protein restriction and is associated with pronounced increases in circulating FGF21 (typically ∼4–6-fold) [79,91,136]. In humans, by contrast, transitions from habitual diets to low-protein diets (<10% P) generally result in relatively smaller increases in circulating FGF21, most often in the range of ∼150% to 300% [91,119,127,128].

The impact of acute increases in circulating FGF21 concentrations on preference for dietary protein is not yet well studied in human trials. Nonetheless, GWAS studies have shown that the variant rs838133 in the FGF21 locus is significantly associated with dietary patterns characterized by low protein intake, high carbohydrate consumption, and low-fat intake [86,88]. Recently, GWAS analysis of the subjects from the United Kingdom Biobank cohort revealed distinctive associations of rs838131 with plasma FGF21 levels and rs838133 with macronutrient intake [131].

## Possible Significance of FGF21 in Early Life

### Rodent studies

Compared with adults, the nutritional regulation of FGF21 levels and its impact on macronutrient preference are less studied in early life. Evidence suggests that lipids in breast milk play a vital role in inducing FGF21 expression in infancy. In newborn mice, hepatic FGF21 gene expression was found to be increased within hours after birth and plasma FGF21 concentrations steadily increased to reach levels higher than those of adult mice by postnatal day (PND)-2, declining thereafter to levels similar to adults by weaning around day-21 [137]. Importantly, initiation of suckling was crucial for this response as mice not allowed to suckle did not show an increase in hepatic FGF21 mRNA expression [137]. The postnatal plasma FGF21 surge paralleled a rise in circulating free fatty acid concentration. This required PPARα signaling, and it could be reproduced by injecting a PPARα agonist or feeding pups a triacylglycerol emulsion [137]. Feeding a glucose solution, however, did not increase FGF21 levels in neonatal mice while the role of proteins and amino acids in driving or sustaining FGF21 levels was not investigated [137]. Whether lipids activate FGF21 signaling exclusively in very early infancy or continue to stimulate FGF21 production throughout infancy remains to be determined. With the rising FGF21 concentrations, there was a concomitant increase in the expression of thermogenic genes in BAT, suggesting that FGF21 may act to switch on thermoregulation in the newborn [137]. This would be of significance in preventing neonatal hypothermia and associated complications [138].

In addition to lipids, protein in breast milk could possibly influence FGF21 expression and metabolic phenotype of infants. The results of a mouse study suggest that protein supply in fetal life and infancy has long-term programming effects on weight gain, appetite, preference for protein (“protein intake target” [139]), and circulating FGF21 concentrations [140]. In this study, dams were fed either a high (35%) or a low (10%) protein diet, starting before mating and continuing during gestation and lactation. The results of this study showed that pups of high-protein-fed dams were heavier at weaning, and they continued to weigh more in adulthood when maintained on a standard chow diet until the age of 46 wk [140]. This was accompanied by an increased intake of daily calories and protein [140]. In food choice experiments, adult offspring of high-protein-fed dams preferred diets with a higher protein content and had significantly lower circulating FGF21 concentration than their counterparts born to low-protein-fed dams [140]. Besides lipids and proteins, the finding that consuming fermentable fibers increases FGF21 concentrations in adult rodents [79,81] raises the question of whether specific types of fermentable oligosaccharides present in human milk (HMOs) [141] could interact with the FGF21 pathway. Emerging evidence from human adults suggests that this might well be the case [142]. Another interesting and relevant observation is the colocalization of *fgf21* and *fut1* genes (important for HMO synthesis) on chromosome 19 [101].

Weaning represents a major transition of a mammalian infant from mother’s milk [or infant formula (IF)] as the sole source of nutrition to an increasing contribution of other complementary sources of nourishment. In rodents, the contribution of dam milk to the pup’s diet starts to decline around PND 14 to 18, becoming minimal between days 21 and 28 as the pup switches to solid foods. This transition results in marked changes in dietary macronutrient balance and type [143,144]. A rat study examined the effects of standard weaning at 3 wk compared with delayed weaning at 4 wk, followed by exposure to an obesogenic HFD until 18 wk of age [145]. The results showed that delayed weaning led to reduced weight gain, lower fat mass, improved insulin sensitivity, and enhanced glucose tolerance on the HFD [145]. These effects were mediated by a sustained increase in plasma FGF21 into adulthood, augmented signaling of FGF21 in GABAergic hypothalamic neurons, and increased thermogenesis in BAT [145]. The extended exposure to macronutrients and bioactive molecules (e.g., milk oligosaccharides) in maternal milk, which are distinctly different from the solid diet, could be a possible driver of these outcomes. It will be important to investigate if the benefits of delayed weaning could be replicated by altering the macronutrient balance of the foods and diets introduced at weaning and by continued exposure to nutrients and bioactives (e.g., milk oligosaccharides) present in breast milk.

The observation of FGF21-mediated long-term effects of delayed weaning in rats on metabolic phenotype indicates possible epigenetic regulation of *fgf21* expression. Indeed, Yuan et al. [146] showed that activating PPARα signaling in the liver of neonatal mice via suckling or with a pharmaceutical ligand of PPARα (Wy14643) caused demethylation of the FGF21 DNA in the liver, leading to an increase in serum FGF21 concentrations (the greater the demethylation, the higher the levels of FGF21). Importantly, suckling led to substantial FGF21 DNA demethylation, and it was further increased by ∼14% in Wy14643-exposed mice. Demethylation status stabilized by the age of 4 wk, remaining largely unchanged thereafter into adulthood (14 wk) [146]. The increase in FGF21 demethylation induced by Wy14643 was dependent on the timing of exposure as treating lactating dams during PND2 to PND16 resulted in significantly greater demethylation, but directly administering Wy14643 to pups for 2 wk at the age of 4 to 6 wk did not affect their demethylation status [146]. This timing-dependent demethylation indicates the possibility of a link between activation of PPARα by milk-derived lipids (such as palmitic acid, oleic acid, arachidonic acid, and docosahexaenoic acid), activation of fat oxidation genes in postnatal life, and epigenetic regulation of FGF21 [146,147]. Importantly, the increased demethylation in early life led to greater responsiveness of FGF21 gene expression when challenged by fasting in adulthood [146]. The Wy14643-induced increase in FGF21 resulted in reduced weight gain in mice when fed a HFD from 4 to 14 wk of age and improved their glucose tolerance [146]. The clear evidence of epigenetic regulation of FGF21 expression highlights the importance of advancing our understanding of the key nutritional regulators of FGF21 expression in infants. This is particularly crucial given that the mass of thermogenic tissue (BAT) in infancy and childhood is linked to a reduced risk of obesity in early life and beyond [146,148,149]. Collectively, this underscores the potential significance of metabolic programming of the FGF21-metabolic health axis for long-term health outcomes.

### Human studies

In humans, FGF21 has been detected in both amniotic fluid and cord blood, and its levels are associated with fetal and postnatal growth; however, the pattern of association varies depending on whether FGF21 is measured in amniotic fluid or cord blood and whether the outcome is birth weight or postnatal growth [62,150,151]. For example, a study showed that FGF21 concentrations in second trimester amniotic fluid have a U-shaped association with fetal growth, where both small for gestational age (SGA) and large for gestational age fetuses have higher FGF21 concentrations than appropriate for gestational age (AGA) fetuses [150]. Furthermore, in infants of females with NGT, cord blood FGF21 (cbFGF21) levels were positively associated with maternal FGF21 concentrations as well as with BMI *z*-scores at 12, 24, and 48 mo of age [62]. However, cbFGF21 levels did not differ between term and preterm infants or between SGA and AGA infants [151].

Consistent with the observations in mice, human infants show a surge in FGF21 levels after birth. FGF21 concentrations were found to be very low in cord blood of healthy breastfed term infants, but the levels increased by almost 5-fold ∼35 h after birth and increased further at the age of 4 mo [152]. Furthermore, FGF21 levels at 4 mo were inversely associated with fasting insulin and HOMA-IR values [152]. Aligned with preclinical evidence on the role of FGF21 in bone homeostasis, FGF21 levels were found to be significantly associated with the length of infants. For example, in a study involving both term and preterm infants, it was found that FGF21 is negatively associated with linear growth. The change in length SD score (SDS) between 0 and 6 mo was negatively correlated with the change in serum FGF21 concentrations [151]. No significant correlation was observed for the change in weight SDS [151]. Term infants with a rapid (>0.67 SDS) increase in length in 0 to 6 mo had significantly lower FGF21 concentrations at 6 mo than those with <0.67 SDS increase [151]. Similarly, in very preterm infants (median gestational age at birth of 28 wk), Guasti et al. [153] showed that FGF21 levels progressively increased in the first 5 wk after birth, and mean FGF21 concentrations during this period were negatively associated with change in SDS for length. No significant association between FGF21 and change in weight SDS was observed in this case either [153]. In these association studies, residual confounding cannot be ruled out, statistically significant associations do not, per se, imply causality, and mechanistic insights into the FGF21-linear growth connection are currently lacking. Other important questions relevant for future investigation in human infants include the impact of duration of breastfeeding and nutritional balance of foods introduced at weaning on the FGF21-metabolic health axis.

Beyond infancy, FGF21 has been found to be associated with BMI and other metabolic parameters in children with various ages. In 6 to 10-y-old children with obesity (BMI >95th percentile), the levels of FGF21 were found to be ∼30% higher than in children with a healthy BMI (BMI >15th to <85th percentile) [154]. Interestingly, within the subgroup of children with obesity, the FGF21 levels were negatively associated with BMI [154]. In another study, circulating FGF21 levels correlated significantly with BMI in children aged ∼10 to 14 y [155]. Others have reported 2-fold higher serum FGF21 concentration in 8 to 16-y-old children with obesity (compared with healthy children without obesity) [156], and children with metabolic syndrome had ∼2.5-fold higher serum FGF21 levels than children without metabolic syndrome [157]. In children with obesity participating in a lifestyle intervention program, the decrease in FGF21 levels correlated significantly with a decrease in SDS-BMI after 1 y [155]. FGF21 was also found to correlate positively with HOMA-IR in 7 to 14-y-old Korean children [158] and with liver steatosis in 5 to 18 y old Taiwanese children [159]. These associations of FGF21 with BMI and metabolic syndrome warrant mechanistic studies to establish whether they arise from FGF21 resistance or merely reflect an epiphenomenon.

Apart from BMI, pediatric studies have demonstrated significant associations of FGF21 levels with height and linear growth. In short-statured prepubertal children with GH deficiency, FGF21 levels were higher than in controls [160]. During the 12 mo of GH treatment, the growth velocity was inversely associated with baseline FGF21 concentrations [160]. Similarly, another study in short-statured prepubertal children (mean age of 5.6 y) showed a significant negative association between serum FGF21 levels and height *z*-score in females [161]. In healthy children without obesity (mean age of 13.5 y), serum FGF21 levels correlated negatively with height SDS [162]. Importantly, FGF21 concentrations in healthy participants and participants with obesity did not significantly associate with bone mineral density [162]. At a molecular level, it was observed in cultured primary chondrocytes (donated by pediatric patients) that FGF21 inhibited GH signaling and GH-induced chondrocyte proliferation and reduced GH-induced IGF-1 expression [153]. These observations in infants and children, when considered alongside rodent literature, point toward a potential role of FGF21 in modulating the balance between linear growth and metabolic health. This possibility should be evaluated in detail in direct mechanistic studies in animal models. Furthermore, as in mice, epigenetic mechanisms may influence long-term FGF21 expression in humans. This notion is supported by the finding that hepatic FGF21 DNA was less demethylated in human fetuses than in adults, but the precise timing of any changes in methylation is not clear [146].

### Does protein intake in early life influence FGF21 physiology?

#### Early protein hypothesis

**FIGURE 3:**
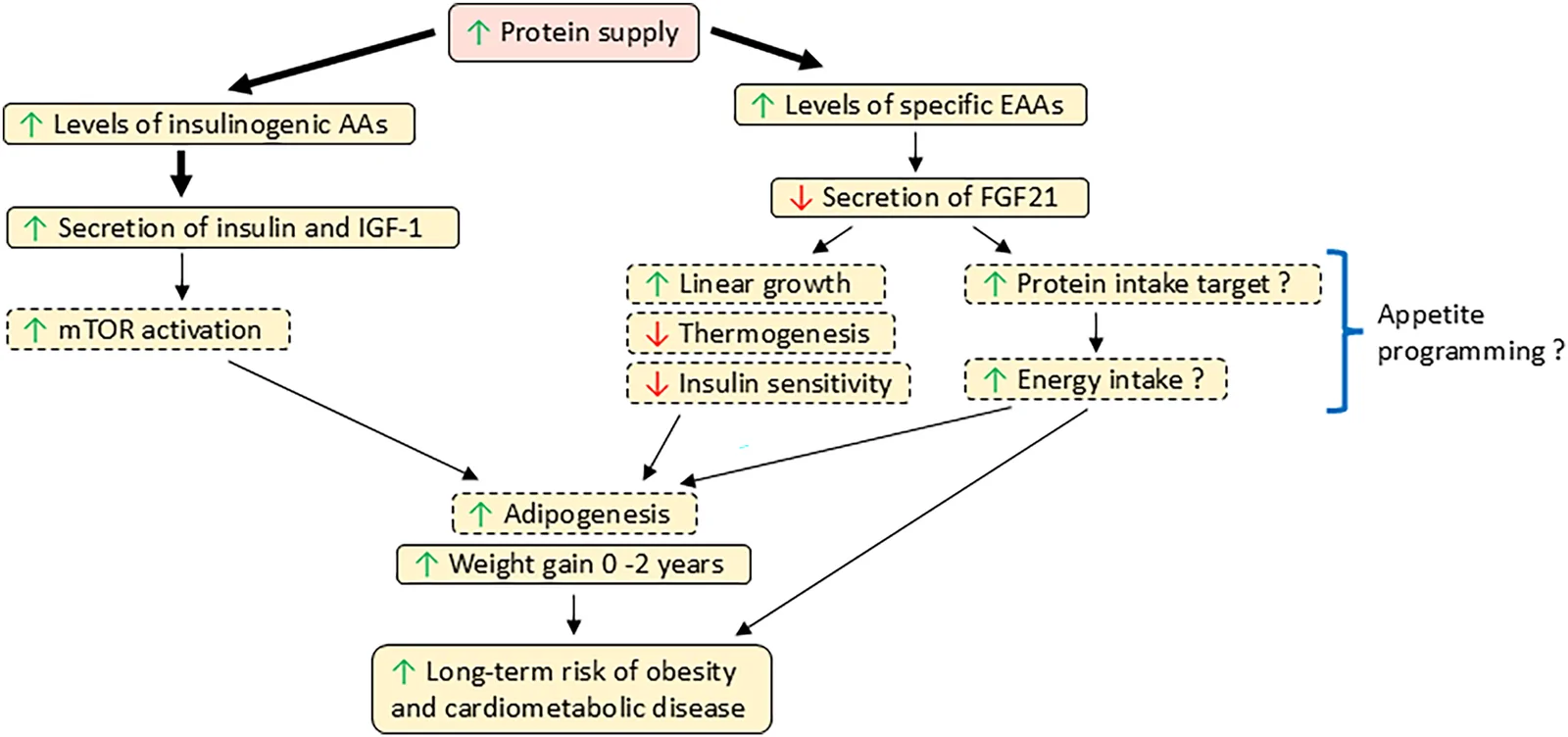
Integrated early protein hypothesis. A higher protein intake during infancy may alter the balance between insulin-like growth factor-1 (IGF-1), insulin, and fibroblast growth factor 21 (FGF21). Elevated protein supply could stimulate insulin and IGF-1 production, potentially enhancing mTOR signaling and thereby promoting increased weight gain and adiposity. At the same time, high protein intake may lower FGF21 levels, which could reduce insulin sensitivity and thermogenesis and possibly accelerate linear growth. High-protein exposure might also program a higher protein appetite set point, contributing to greater energy intake later in life. Collectively, these interacting pathways may increase risk of obesity and related metabolic disorders in later life. Thicker arrows indicate effects of high-protein intake observed in human infants, whereas thinner arrows represent hypothesized effects derived from human association studies and preclinical evidence. AA, amino acid; EAA, essential amino acid; mTOR, mechanistic target of rapamycin; ?, yet to be directly investigated. Yellow boxes with solid outline: findings from human infants. Yellow boxes with dashed outline: hypothesized effects.

The early protein hypothesis (EPH) centers on the concept of metabolic programming by dietary cues and proposes a link between excess protein intake during early life and increased long-term risk of obesity and associated disorders (Figure 3) [163]. The EPH hypothesizes that a high protein intake exceeding an infant’s needs results in elevated plasma concentrations of insulinogenic essential amino acids, especially BCAAs [164]. These amino acids stimulate the secretion of insulin and IGF-1, key growth factors that are well-established activators of mTOR [163,164]. The activation of mTOR in response to BCAAs, particularly leucine, would drive enhanced weight gain and fat deposition in early life (0–2 y) [[163], [164], [165]]. This is also thought to contribute to a long-term susceptibility to obesity and potentially related health issues via metabolic programming [163,164].

The EPH is supported by results of studies showing greater weight gain in infants fed formulas with a higher protein content compared with the reference group of breastfed infants. This may be due to the relatively higher protein content of IF compared with breast milk. Additionally, the protein content of breast milk progressively declines with advancing phases of lactation, whereas it remains relatively static in IF [163,164,166,167]. Importantly, in several clinical trials, it has been found that reducing the protein content of IF brings the growth trajectory of IF-fed children closer to breastfed children [[168], [169], [170], [171]]. The most notable example is the multicenter European Childhood Obesity Program (CHOP) study that enrolled >1500 infants [168]. In this study, infants were randomly assigned to receive an IF with either a lower protein content (1.8 g protein/100 kcal) or a higher protein content (2.9 g protein/100 kcal), with protein derived from cow milk, during the first 4 to 5 mo of life [168]. Afterward, infants in the low-protein group received a follow-on formula (FoF) containing 2.2 g protein/100 kcal, whereas those in the high-protein group received an FoF containing 4.4 g protein/100 kcal [168]. The researchers compared the growth of these infants with that of a breastfed reference group and discovered that those who consumed the higher protein IF (and FoF) had higher BMI and higher *z*-scores for weight and length at 24 mo of age [168]. Infants maintained on the low-protein IF (and FoF) had significantly lower BMI than the high-protein group and their growth trajectory was relatively more similar to that of breastfed infants [168]. Other studies have also reported increased body weight [169], weight gain [170], fat-free mass index [172], and bone mineral content (not significantly higher when normalized to lean mass) [170] in infancy with a higher protein IF. Follow-up of children in the CHOP trial at 6 y of age showed that BMI in infants fed high-protein formulas was significantly greater than that in those fed low-protein formulas, and the differences became more pronounced at the higher percentiles of BMI [173]. The odds of obesity in the high-protein group were 2.4 times higher than that in the low-protein group and 2.8 times higher than that in the breastfed infants [173]. Besides BMI, fat mass index and fat-free mass index estimates at 2 to 6 y of age were higher in children in the high-protein group [174]. Overall, this study showed that increased rate of growth in infancy may have long-lasting metabolic consequences. Indeed, rapid weight gain in early infancy is linked to a higher risk of childhood obesity [[175], [176], [177]]. For example, the meta-analysis by Druet et al. [175] of individual growth trajectories found that each unit increase in weight SDSs during the first year doubled the risk of childhood obesity and increased the risk of adult obesity by 23%. Similarly, analysis of the Boston Birth Cohort data revealed a 50% increased risk of being overweight/obese by the age 2 to 7 y for each SD increase in weight gain during the first 4 postnatal mo [176]. These findings highlight the importance of optimizing weight gain in infancy for long-term metabolic health benefits.

Here, it is important to note that although the CHOP study showed a clear benefit of reducing the protein content on the growth trajectory of infants, results of recent meta-analyses suggest that the benefit of reducing IF protein content on reducing rapid pace of growth are not observed consistently across studies [178,179]. In addition, a meta-analysis by Gale et al. [180] reported a modest yet paradoxical increase in fat mass in breast-fed compared with infant formula (IF)–fed infants at 3 to 4 and 6 mo of age (mean difference: 0.18 kg). This difference may reflect nutritional factors other than protein, such as the higher sn-2 palmitate content of breast milk [181,182]. By 12 mo of age, however, fat mass was higher in IF-fed infants (0.29 kg), whereas fat-free mass was consistently greater in IF-fed infants throughout infancy [180]. It is also important to note that the CHOP study included differences in body weight at baseline between the breastfed reference group and the IF-fed groups, infants were enrolled over a relatively broad age range (0 to 8 wk), and participants were switched to FoF with low and high protein content relatively early (around 5 mo of age) [168,173]. The study found that high protein IF was specifically detrimental to BMI in children at the age of 6 y who were growing above the 90th percentile compared with the children at the 50th percentile [173]. Although the median, 85th, and 90th BMI percentiles were higher in the high-protein group, only the 95th percentile differed significantly between groups. Obesity prevalence in the whole cohort was low (5.8%), with only 6 children (2.6%) in the LP group compared with 17 children (7.7%) in the HP group exceeding the cohort-specific 95th BMI percentile, corresponding to a modest difference of 5.6% in the prevalence of obesity between the high-protein and low-protein groups. Therefore, additional evidence from larger studies is needed to support the broader generalization of the CHOP trial findings.

What is clear from the literature is that despite lowering the protein content of IFs, the weight gain in IF-fed children continues to be greater than that of breastfed infants, especially at 4 to 6 mo of age [183,184]. In addition, in several IF studies, data for circulating insulin and IGF-1 show higher levels of these anabolic hormones in IF-fed (including low-protein IF) than breastfed infants [169,[185], [186], [187]]. One of the possible reasons for this could be the existent differences in the amino acid profile of IFs and breast milk. Modern-day IFs are mostly manufactured using cow milk protein and are whey dominant to mimic the whey-casein ratio of breast milk [166]. CHOP study is an exception as it used casein-dominant IFs [168]. Of note, the content of amino acids in breast milk progressively declines with the stage of lactation, whereas the regulations for minimum levels of essential amino acids in IF remain constant [[188], [189], [190]]. Consequently, even the low-protein IFs often have higher amounts of certain essential amino acids such as BCAAs (leucine, isoleucine, and valine), lysine, methionine, and threonine than breast milk [169,[186], [187], [188], [189],^191^]. Therefore, to make the growth trajectory of IF-fed infants more consistently similar to that of breastfed infants, further optimizing the amino acid profile of the protein supplied in IF represents a possible next step. This could potentially reduce the gap between breastfed and IF-fed infants in terms of their weight gain profile and associated metabolic hormonal signature (insulin, IGF-1, and FGF21). Beyond their effects on weight gain and circulating metabolic hormones, differences in protein quantity and amino acid composition during infancy may influence additional aspects of metabolic programming. Future clinical and preclinical studies should therefore include comprehensive assessment of developmental and metabolic traits that could be shaped by endocrine pathways involving IGF-1, FGF21, and other hormonal mediators, including their effects on the development and long-term function of key metabolic tissues such as adipose tissue, skeletal muscle, liver, pancreatic islets, and the central regulation of energy balance.

#### Relevance of FGF21 for early protein hypothesis

Considering the evidence that protein and amino acid intake play a major role in modulating FGF21 physiology and that FGF21 in turn stimulates protein intake, we propose to integrate FGF21 as a possible mediator in the EPH (Figure 3). Our aim here is to provide a scientific framework for generating testable hypotheses for future mechanistic studies rather than drawing definitive conclusions based on the very limited available data. Following this approach, in addition to increasing anabolic hormones (insulin and IGF-1), a high protein supply in infancy could suppress the levels of FGF21. The postulated decrease in FGF21 levels would result in lower fat oxidation, reduced thermogenesis, decreased insulin sensitivity, increased linear growth, and greater weight gain. In such a theoretical model, FGF21 will act as a counterbalance to insulin and IGF-1, similar to the well-described insulin-glucagon balance in metabolic regulation, and a high protein supply would tilt this balance toward greater weight gain and linear growth in the first few months of life. On the basis of observations in mice, we anticipate these effects to be more noticeable in males [110]. Preliminary supportive evidence for our hypothesis comes from early data from a recent study showing that the protein content of IF does indeed influence FGF21 levels [192]. The study looked at hormone levels in infants who were fed either a low-protein or high-protein IF (1.85 compared with 2.50 g protein/100 kcal) in the first month after birth. Both groups then continued with the 1.85 g IF until the age of 3 mo and then switched to a 1.50 g protein FoF until the age of 12 mo. Preliminary results show that FGF21 levels were more responsive to differences in the protein content of IFs than insulin and IGF-1, and levels of FGF21 across the 12 mo after birth were relatively higher in breastfed than in IF-fed infants [192]. Importantly, infants receiving higher protein IF had significantly lower FGF21 levels after 1 month compared with those receiving lower protein IF, a trend that persisted ≤4 mo, and the differences in FGF21 levels were more substantial in males [192]. Whether and how differences in FGF21 levels during infancy influence weight gain and linear growth remain to be fully elucidated. Moreover, although evidence from adult human and rodent studies suggests that protein is the primary nutritional regulator of circulating FGF21 concentrations [103,126,130], it remains unclear whether other nutrients—particularly dietary fat—also exert sustained effects on FGF21 concentrations in early life. It has been proposed that reduced fat intake during early life may lower circulating leptin levels and could program individuals toward a long-term “thrifty” metabolic phenotype; such early life fat restriction has also been suggested as a potential contributor to the rising incidence of childhood obesity [193]. This concept becomes quite relevant during the transition from exclusive milk feeding to the introduction of relatively low-fat complementary foods at weaning. In this context, it would be worth exploring if FGF21 serves as a key node linking the effects of protein and fat intake in early-life with metabolic programming.

Preclinical evidence suggests that the decrease in FGF21 concentrations in early life could have long-term consequences for cardiometabolic health and appetite regulation. For example, high protein intake in infancy could perhaps program the FGF21 DNA methylation status and increase the set point for protein appetite in later life [140,146]. In addition, this could also affect long-term preference for carbohydrates and sweet taste and metabolic response to specific fatty acids [2,5]. Together, this would potentially increase the susceptibility to obesity and likely induce greater calorie intake on diets consumed during childhood and adulthood. Supportive evidence for these proposed mechanisms is currently lacking, and future studies are warranted to test these hypothesized effects.

Beyond protein quantity, the integrated EPH would also facilitate investigating the consequences of protein quality, notably the non-BCAA essential amino acids. From preclinical studies, it is evident that compared with BCAAs, dietary restriction of other essential amino acids such as threonine, lysine, and methionine more strongly increases FGF21 levels [[118], [119], [120], [121],^123^]. Therefore, if these non-BCAA amino acids are higher in IF than breast milk, they could mask some of the FGF21-related benefits expected from reducing the IF protein content. To explain this point, we take the ProtEUs clinical study as an example. In this study, the investigators compared the growth and metabolic outcomes in infants fed an IF with either a lower (1.7 g protein/100 kcal) or higher protein content (2.1 g protein/100 kcal) from birth to 6 mo of age [172,185]. The amino acid profile of the lower protein IF was based on a series of studies using the indirect amino acid oxidation (IAAO) technique to calculate the essential amino acid requirements of infants in the first month of postnatal life [[194], [195], [196], [197], [198], [199]]. Interestingly, the calculated requirements for FGF21-relevant amino acids such as methionine and lysine were substantially higher than the amounts consumed by the breastfed infants, whereas the calculated requirement of tryptophan was ∼50% lower [[197], [198], [199]]. The results of this study showed that weight gain after 4 mo of IF-feeding was similar between the 2 IF groups and was significantly greater than that of breastfed infants [172,185]. It is possible that the essential amino acid estimates derived from IAAO methodology are driven mainly by what is needed for efficient and maximal tissue accretion. Indeed, it has recently been suggested that the IAAO methodology estimates protein and amino acid needs aimed at maximizing anabolism, rather than requirements for nitrogen balance maintenance [200]. In contrast, breast milk seems to restrict the supply of specific amino acids below the maximum “requirement,” and this could potentially serve as a signal to induce FGF21 production in the infant. Apart from stimulating fat oxidation and improving insulin sensitivity, increased circulating levels of FGF21 may contribute to sustaining BAT activity and maintaining beige adipocyte reserves in breast-fed infants [2,137,145]. In such a system, the higher tryptophan levels in breast milk possibly act to increase brain serotonin levels, thereby inducing satiety and keeping in check any FGF21-driven excess protein and calorie intake [107,201]. Investigating these proposed physiological pathways would require examining how the amounts of protein consumed during infancy and its amino acid composition affect FGF21 production and its metabolic effects.

## Important Questions for Future Research

To further understand the role of FGF21 in infant growth, development, and metabolic programming, we recommend routinely measuring FGF21 (in addition to IGF-1 and insulin) in clinical trials involving IF and breastfed infants. This would provide invaluable information about how nutrients such as proteins, amino acids, bioactive lipids, HMOs, etc., influence FGF21 status in early life and how this relates to postnatal growth trajectory. This effort should be complemented by direct assessment of the effects of experimentally modulating circulating FGF21 levels in early life—through nutritional or pharmacological interventions—on weight gain, body composition, linear growth, markers of metabolic status, and epigenetic programming in preclinical models. In the same vein, the observed links between FGF21 concentrations in maternal blood, amniotic fluid, and cord blood—and their relationship to early childhood growth [62,150,151]—highlight the critical need to investigate how maternal nutrition may shape the interplay between FGF21 and offspring development.

**FIGURE 4:**
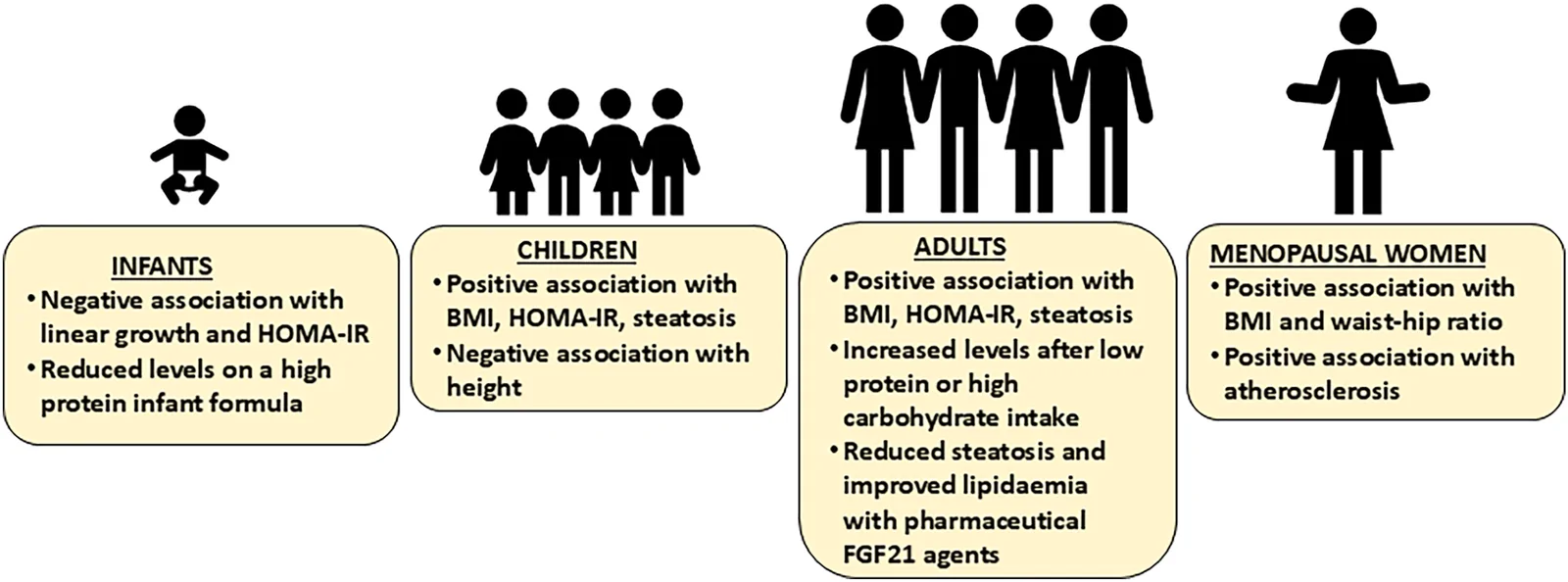
Summary of the relationship between fibroblast growth factor 21 (FGF21) and metabolic parameters in humans at various stages of life. Associations shown are between circulating FGF21 concentrations and health parameters. Pharmaceutical agents include FGF21 analogs and receptor agonists. In infants, FGF21 is negatively (inversely) associated with linear growth. In children, adults, and females with menopausal age, FGF21 is positively associated with BMI. Reducing protein intake strongly induces FGF21 production in infants and adults. FGF21-based pharmaceutical agents have been shown to improve blood lipid profile and reduce hepatic fat content in adults. HOMA-IR = Homeostatic Model Assessment for Insulin Resistance.

Another area requiring further investigation is how the carbohydrate-rich foods and diets introduced at weaning alter FGF21 signaling in the child. This would inform the composition of complementary foods that could assist in mimicking the FGF21-related benefits of delayed weaning. There is also a need for a better understanding of the role of FGF21 in the development of appetite signaling in the brain. Emerging evidence suggests that weight gain in early life could influence satiety set points [202,203], but the specific role of protein–FGF21 axis on long-term satiety responsiveness in humans remains to be investigated. Overall, future research should explore the short- and long-term consequences of nutritional modulation of FGF21 levels in infancy on its physiological functions, sensitivity to dietary cues, and subsequent influence on macronutrient preferences, satiety signaling, and caloric intake in childhood and adulthood. In this context, it will be critical to determine the relevant time window for beneficially modifying FGF21-mediated early metabolic programming processes. For these objectives, the proposed EPH, which integrates potential FGF21-related pathways and mechanisms, aims to provide a scientific framework for generating new hypotheses for experimental testing. Altogether, a deeper understanding of FGF21 biology in early life promises to provide long-term advantages for health- and lifespan (Figure 4).

## Author contributions

The authors’ responsibilities were as follows – JAW, JMR-N: conceptualized the topic and wrote the manuscript; AB, EMvdB: edited and reviewed the manuscript; and all authors: read and approved the final manuscript.

## Declaration of Generative AI and AI-assisted technologies in the writing process

During the preparation of this work, the authors used ChatGPT in order to improve readability and language. After using this tool/service, the authors reviewed and edited the content as needed and take full responsibility for the content of the publication.

## Funding

The authors reported no funding received for this study.

## Conflict of interest

All authors are employees of Société des Produits Nestlé S.A.
